# Radionuclides distribution and radiation hazards assessment of black sand separation plant’s minerals: a case study

**DOI:** 10.1038/s41598-024-55633-1

**Published:** 2024-03-04

**Authors:** Islam M. Nabil, Moamen G. El-Samrah, A. F. El Sayed, Ahmed Shazly, Ahmed Omar

**Affiliations:** 1 Physics Department, Faculty of Science, Fayoum University, Fayoum, Egypt; 2 Nuclear Engineering Department, Military Technical College, Kobry El-Koba, Cairo Egypt; 3 Physics Department, Faculty of Science, Cairo University, Cairo, Egypt; 4 Central Laboratory, Cairo, Egypt

**Keywords:** Black sand, Separated products, NORMs, Activity concentrations, Radiological hazard indices, Applied physics, Nuclear physics, Techniques and instrumentation, Environmental impact

## Abstract

This study assessed the radioactivity levels and associated risks in the black sand-separated products obtained from the black sand separation plant in Delta, Egypt. A total of sixteen samples were taken from hot spots during and after the separation process. These include water samples and other samples that represent monazite, rutile, zircon, granite, ilmenite, and silica products. The hot spots included the area where the ore was stored. The activity concentrations of $^{232}Th$, $^{226}Ra$, and $^{40}K$ were determined in these samples using a p-type HPGe detector. Based on gamma spectrometric analysis, samples of rutile, zircon, and monazite had the highest amounts of radioactivity because they contained the highest NORM’s activity concentrations. In addition, it indicated that the radiological hazard indices of the collected samples were higher than the average world limits for sand texture. These findings suggest that the black sand separation process reveals potential risks to human health and the environment, and therefore, appropriate measures need to be taken to mitigate these risks, especially for the safety of the workers on-site. Reducing the risk associated with those sites should be controlled by implementing the recommendations declared for the series of International Basic Safety Standards of the International Atomic Energy Agency (GSR) Part 3, as affirmed in Document No. 103 of 2007 by the International Commission on Radiological Protection (ICRP) as will be presented in the paper body.

## Introduction

Living beings are exposed to ionizing radiation from naturally occurring radioactive materials (NORM) or even from radioactive materials that have been technologically enhanced (TENORM), in addition to exposure to artificial radioactive sources^1,2^. Mining and milling activities and other activities attributed to the manufacturing or extraction of phosphate fertilizers and building materials may increase the concentrations of the naturally existing NORMs thus increasing the associated radiological risks^3^. Therefore, this increase can cause people to be exposed to or breathe in radionuclides, exposing them to high levels of ionizing radiation that can be beyond the recommended yearly limits for radiation exposure. Due to TENORM residue in both commercial and industrial products, it can lead to radioactive contamination of soil and water, exposing workers and the general public to radiation exceeding recommended limits. Therefore, it is critical to perform an environmental radiological assessment to understand the possible risks upon exposure to the inherent radiation sources and take the necessary protective measures^4^.

The primary environmental radioactive materials that are usually found in black sand or mineralogical samples are thorium ($^{232}Th$), uranium (U) series, and their decay products, as well as potassium ($^{40}K$)^5^. These elements emit ionizing radiation that can cause various harmful effects on living cells, which are usually chronic in the form of possible mutations or increasing the risk of cancer in the long term if their concentrations exceed the recommended global limits^6^. Black sand has different mineral compositions globally due to geographical factors^7,8^. The valuable heavy minerals in black sand are extracted after many physical, mechanical, and electrostatic processes^9–12^. The extracted minerals from the black sand plants (e.g., ilmenite, rutile, magnetite, monazite, zircon, silica, and granite) have many uses in various industrial fields^13^. The minerals extracted from black sand are used in various industries, from the ceramic industry to building materials and automobile industry to various electronic and technological industries (e.g., sanitary ware, glasses, mineral fertilizers, water filters, electronic chips, space technology, and shielding material)^14–16^.

Kotb^17^ investigated the hazard indices related to the radiological evaluation of raw monazite material in different grades (90$\%$, 75$\%$, and 50$\%$). The $^{232}$Th, $^{238}$U, and $^{40}$K activities were calculated. It was concluded that there was an overage of 20 mSv.y$^{-1}$ in the calculated effective and absorbed dosages for the public which exceeds the recommended 1 mSv annual dose. Hence, radiation safety measures must be implemented to reduce the risks^18^. Other researchers^19^ evaluated the intrinsic radiological qualities of black sand samples by studying their chemical composition and activity concentrations of the existing naturally occurring radioactive nuclides and computing the associated radiological hazard indices. Minerals like zircon and rutile had more of their inherent content of NORMs concentration concentrated during the upgrading process. Magnetite and ilmenite, on the other hand, had less than what is considered acceptable^20^. In addition, the risk analysis uncovered potentially harmful circumstances and offered recommendations for mitigating such risks so that the workplace remained safe for the workers^21^.

This study aims to determine the activity concentrations and hazardous radiological indices related to the black sand separation (BSS) process and the yielded separated products, which indicates how they affect the work environment. This was done by determining the activity concentrations of the contained NORMs in all extracted mineral products obtained from the separation processes (ilmenite, rutile, magnetite, monazite, zircon, silica, and granite) in addition to calculating the radiological hazard indices (e.g., absorbed gamma dose rate (D), outdoor and indoor annual effective dose ($E_{out}$, $E_{in}$), radium equivalent ($Ra_{eq}$), internal and external hazard index ($H_{in}$, $H_{ex}$)) which helps in anticipating the BSS process’s potential health risks to workers and any nearby resident public.

## Experimental procedure

### Sample collection

**Figure 1:**
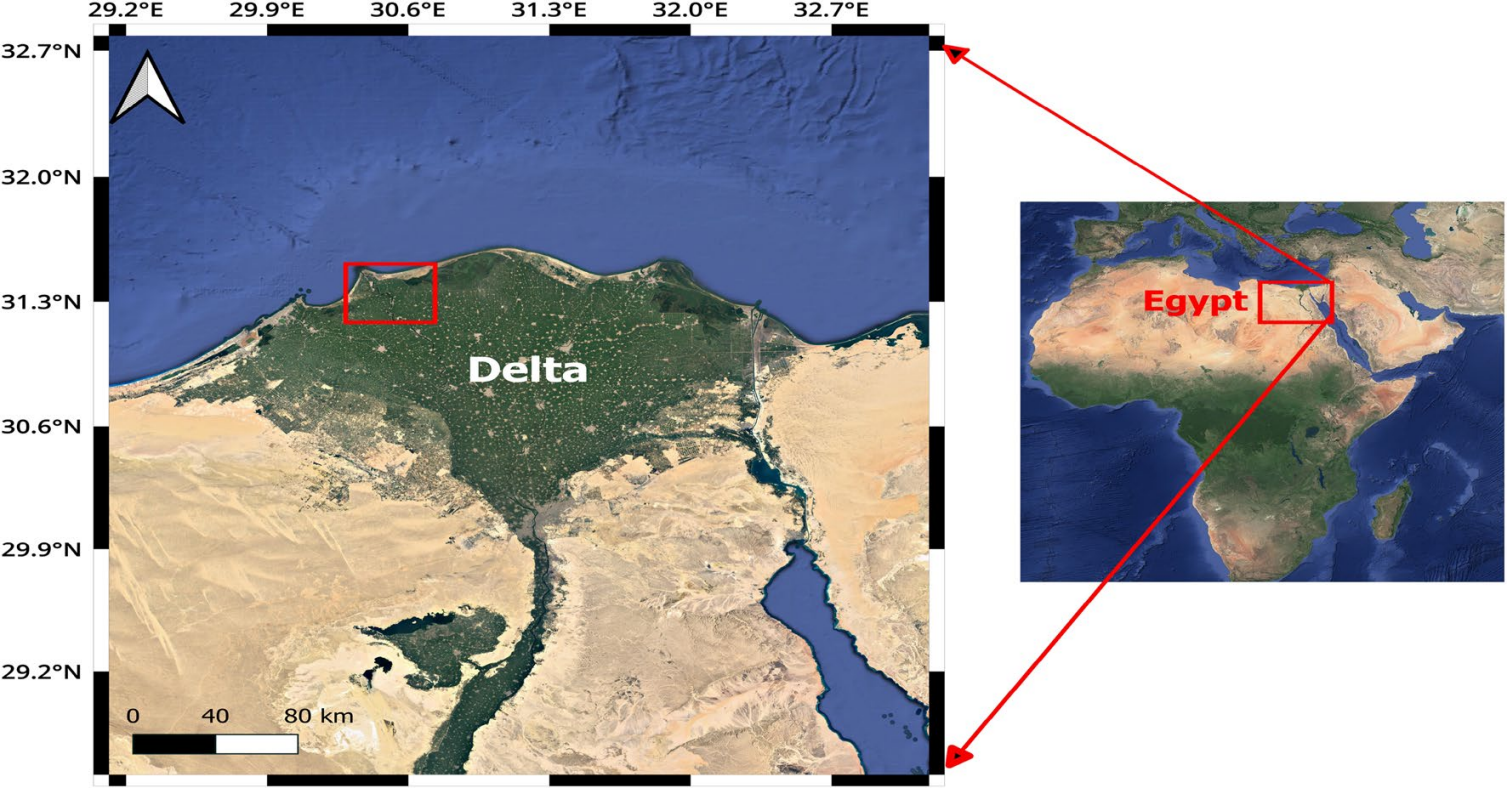
Black sand separation plants’ location, (Using QGIS (V. 3.30.3) software, Image Landsat-Copernicus Data: SIO NOAA, U.S. Navy, GEBCO).

Black sand ore is taken out of the ground by huge dredgers that dig very deep into the ground in certain places, usually on the shores of oceans, seas, or rivers^17^. Black sand separation plants take the ore they’ve extracted and use sequential processes to separate the ores of economic interest along their production lines. These processes include introducing black sand concentrate as a wet sludge and magnetic separation followed by intentional and natural drying via shaking tables and natural hot air, respectively. Twelve sand samples (SS) were collected from the black sand separation plant in the area located on the north shore of the Delta region, as shown in Fig. 1. Four samples were taken from the storage area of the main ore and rutile. One sample was taken from the area that has stacks of garnite, ilmenite, magnetite, silica, and zircon. Three samples were taken from the storage area for monazite. Moreover, four water samples (WS) were taken from the pure supply water lake, the waste water lake, and the shaking tables.

### Sample preparation

The preparing stages of the soil samples begin with the drying stage, where an appropriate amount of each sample (e.g., 400 g) is taken in glass dishes and placed in the drying oven at a temperature approaching 105 $^{\circ}$C, left for 6 h, and then weighed again^22^. This method is repeated several times until the weight of the samples stabilizes. The samples are then ground and sieved to a particle size of < 2$\mu$m. The samples are then filled into a standardized polyethylene Marinelli-beaker container (240 ml), weighed, and the density of the samples adjusted so that each sample can have the same density as a certified standard volumetric multi-source, which is used to calibrate the efficiency of the gamma spectrometer^23,24^. Then, the packed samples were sealed well for 28 days to ensure the secular equilibrium between $^{226}Ra$ and its progenies (the full decay of radon gas $^{222}Rn$, half-life = 3.8 days)^25,26^. This step ensures that the daughters remain enclosed and the radon gas is confined within the volume of the sealed beakers^27,28^. For the collected water samples, a liter of water from each sample was evaporated at 105 $^{\circ}$C until the volume was reduced to a volume of 240 ml (Marinelli beaker volume) in the oven and filled directly into sample containers, which have the same geometry as the authoritative source (Irish seawater IAEA-381)^29,30^.

### Detection setup and gamma-ray spectrometry

**Figure 2:**
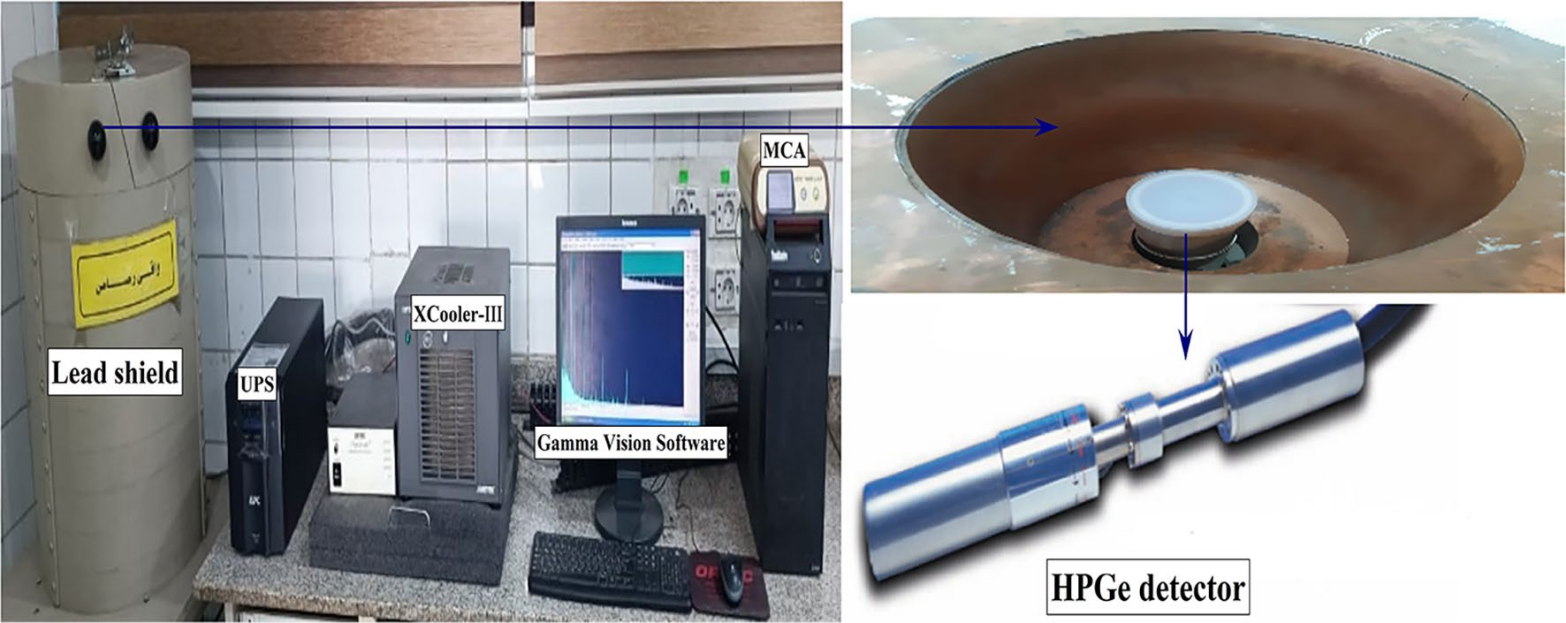
Radiological analysis system used to analyze BSS samples.

A p-type coaxial hyper pure germanium (HPGe) detector has been used to determine the activity concentration of $^{232}Th$, $^{226}Ra$, and $^{40}K$ in the BSS samples. The selection of the HPGe detector was due to its high relative efficiency (100$\%$) in comparison to the NaI(Tl) 3”×3” scintillation detector, which ensures high counting efficiency and, at the same time, takes advantage of the higher resolution of the HPGe semiconductor detector^31^. The detector demonstrates an energy resolution of 1.32 keV concerning the $\gamma$-ray energy of 122 keV. The detector is maintained vertically in a cylindrical lead shield of 10 cm thickness lined with 2 mm Cu foils. The Ge crystal will be maintained at the temperature of liquid nitrogen based on the cooling process through a cryostat model 7500SL. The detector is operated by a high-voltage power supply at +3600 V, coupled with the computer through a built-in preamplifier model 2002 CSL, by which the output signal is connected to a shaping amplifier, followed by a multichannel analyzer (MCA) unit (DSPEC jr.2) as seen in Fig. 2^32^. The output spectrum is analyzed by GammaVision 6.09 software^33^. For all measurements, the live time has been set up to 24 hs, where the dead time will be consistently below 2$\%$ and enhance the accuracy of the radiation measurement process^34^.

**Figure 3:**
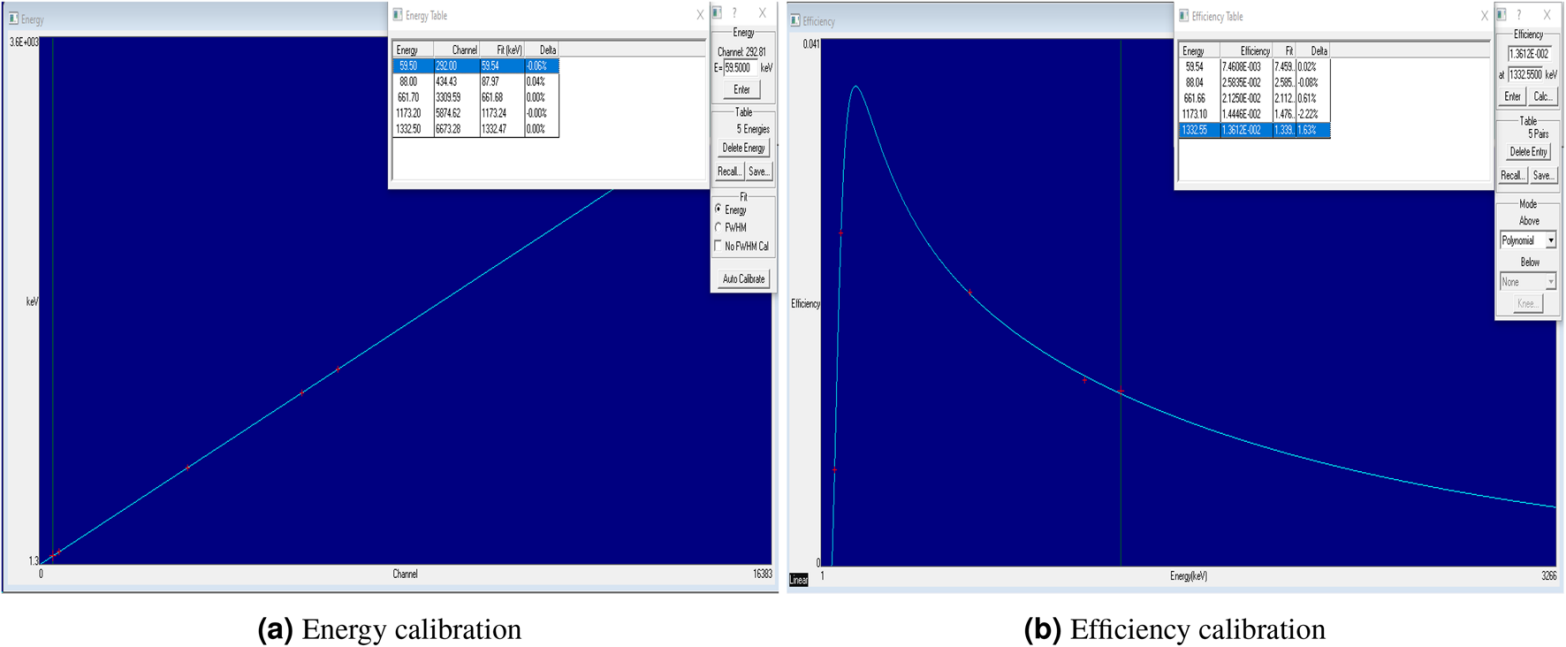
Energy and Efficiency calibration of HPGe detector using Gamma-Vision software.

Finally, the accuracy of the measurements is based on energy and efficiency calibration, detector performance, and the background spectra, which are used to correct the net peak areas of the collected spectra^35^, which was taken into account for the BSS sample material and the certified standard volumetric source material as seen in Fig. 3. Therefore, the energy and efficiency calibration of the detector has been performed using the certified standard volumetric source (240 ml) of epoxy material, which contains radioactive isotopes of ($^{241}Am$, $^{109}Cd$, $^{137}Cs$ and $^{60}Co$). Moreover, the radioactivity of this standard source has been updated, depending on the production date and the initial activities with their uncertainties (U) in Bq^36,37^. The difference between the measured sample matrix (chemical composition) and the used standard volumetric source material was taken into account through the analysis software (Gamma Vision 6.09) and according to previously published methods in this regard^38^.

The investigated BSS samples were measured for the $^{226}Ra$, $^{232}Th$ and $^{40}K$ radionuclides. The activity concentration of $^{40}K$ was measured directly via its $\gamma$-ray energy of 1460.83 keV (10.70$\%$). However, the activity concentration of $^{226}Ra$ was determined based on the average values of $\gamma$-ray transitions of its daughters, which are $^{214}Pb$ with emitted $\gamma$-rays of 295.10 (19.20$\%$) and 351.90 (37.10$\%$) keV and $^{214}Bi$, which emits $\gamma$-ray energies of 609.30 (46.10$\%$), 1120.28 (15.90$\%$), and 1764.50 (15.90$\%$) keV. On the other hand, the activity concentration of $^{232}Th$ series has been identified due to the average values of $\gamma$-ray energies of 238.60 (43.60$\%$) keV emitted from $^{212}Pb$, and 338.40 (12.00$\%$), 911.10 (29.00$\%$) and 968.90 (17.40$\%$) keV from $^{228}Ac$, in addition to 583.10 (86$\%$) and 2614.00 keV (99.10$\%$) emitted from $^{208}Tl$^39^.The quality control and quality assurance of the results of this study are based on the International Atomic Energy Agency (IAEA) reference materials (Cu-2010, 312, and 375)^40^. The activities of the radionuclides are corrected in consideration of the production date and initial activities according to the following equation:^41,42^,

1 $$\begin{aligned} A=A_0 \times e^{-\lambda t} \hspace{0.5cm}, \end{aligned}$$

where A is the corrected activity for time t, $A_{0}$ is the initial production activity, and $\lambda$ is the decay constant for the particular nuclide. Hence, the activity concentrations $A_{c}$ of the $^{226}Ra$, $^{232}Th$, and $^{40}K$ in the measured samples have been determined based on the following equation, depending on the emitted $\gamma$-ray from the daughter radionuclides^43^.

2 $$\begin{aligned} A_{c} (Bq.kg^{-1}) = \frac{C_{net/secs}}{I_{\gamma }(E)\times \varepsilon _{abs}\times m } \end{aligned}$$

$A_{c}$ is the activity concentration, $C_{net}$ is the net number of counts in a specific peak per second, $I_{\gamma }$(E) is the emission probability of gamma with certain energy per disintegration, $\epsilon _{abs}$ is the photo peak absolute efficiency at a certain energy, and m is the mass of the measured sample (kg).

A Marinelli beaker filled with deionized water was used to estimate the background radiation three times for 24 hours. The Marinelli container has the same geometry applied to the sand samples. The minimum detection activity (MDA) of each radionuclide in a spectrum is calculated from the background spectrum with the same conditions, such as sampling time, geometry, and amplifier gain. The net count for each nuclide is determined by subtracting the background count from the sample count^44^. Then, both the detection limits and the MDA values of the detection system were calculated using the following equations^45,46^:

3 $$\begin{aligned} MDA = \frac{L_{D}}{\varepsilon _{abs}\times I_{\gamma }(E) \times T} \hspace{1cm}, L_{c}=2.32\sigma _{B} \hspace{0.5cm} and \hspace{0.5cm} L_{D}=2.706 + 4.65\sigma _{B}, \end{aligned}$$

where $\sigma$ $_{B}$ is the background counting, and T is the counting time of the sample. The critical level L$_{C}$ is defined as the level above which the net counts present some detected activity with a certain degree of confidence; L$_{D}$ is the detection limit. Table 1 shows the radionuclides and gamma-ray energy peaks; these are mainly due to the $^{238}$U, $^{232}$Th decay chains, and $^{40}$K.

**Table 1:** The MDA for the 100 $\%$ p-type HPGe detection systems.

Series	Nuclide	Energy (Kev)	MDA (Bq)
K-40	K-40	1460.99	0.73
U-238	Th-234	63.00	1.09
	Pa-234m	1001.3	6.18
	Ra-226	185.99	0.04
	Pb-214	295.22	0.23
		351.99	0.15
	Bi-214	609.32	0.16
		1120.28	0.39
		1764.51	0.45
Th-232	Ac-228	338.40	0.38
		911.07	0.22
		968.90	0.22
	Pb-212	238.63	0.10
	Bi-212	727.17	0.75
	Tl-208	583.14	0.067
		2614.47	0.11

### Radiation hazard indices calculations

These indices are used in various radiation protection applications, such as regulatory compliance, environmental monitoring, and occupational exposure control.

#### Radium equivalent ($Ra_{eq}$), internal and external hazard index ($H_{in}$, $H_{ex}$) calculations

Radium equivalent ($Ra_{eq}$), internal hazard index ($H_{in}$), and external hazard index ($H_{ex}$) are all essential measures of radiation safety that are used to assess the potential risks associated with exposure to ionizing radiation from materials that contain naturally occurring radioactive isotopes^47,48^.

Radium equivalent ($Ra_{eq}$) indicates an equivalent value summed the specific activities of the main three naturally occurring radioactive nuclides, $^{238}$U, $^{232}$Th, and $^{40}$K, assuming they are all in terms of $^{226}$Ra-specific activity, knowing that 370 Bq kg$^{-1}$ of $^{226}$Ra, 370 Bq kg$^{-1}$ of $^{238}$U, 259 Bq kg$^{-1}$ of $^{232}$Th, and 4810 Bq kg$^{-1}$ of $^{40}$K yield the same $\gamma$-ray dose rate. The safe value is considered below 370 Bq kg$^{-1}$^49^.

Internal hazard index ($H_{in}$) is an index that considers receiving indoor doses from gamma rays and radon. It depends on the same factors used for estimating H$_{ex}$; however, the effect of $^{226}$Ra is considered twice due to the emitted gamma rays and the internal dose that can be received due to inhalation of radon gas in improperly ventilated places. The value should be below 1 to be in the safe range^50^.

On the other hand, the external hazard index ($H_{ex}$) is used mainly to describe the external radiological hazard that arises from direct and prolonged contact with materials containing appreciated amounts of NORMs like the case of workers who work in mining, separation, and piling up of black sand and its separated products. The safe limit is below unity.

All the abovementioned parameters and indices provide a comprehensive evaluation of the potential risks associated with exposure to ionizing radiation. The following equations are typically used to calculate $Ra_{eq}$, $H_{in}$, and $H_{ex}$^51,52^:

4 $$\begin{aligned}{} & {} Ra_{eq}(Bq.Kg^{-1})= 1.43 \times A_{TH} + A_{Ra} + 0.077 \times A_{K} \end{aligned}$$

5 $$\begin{aligned}{} & {} H_{in}= \frac{A_{Th}}{259} + \frac{A_{Ra}}{185} + \frac{A_{K}}{4810} = \frac{A_{^{222}Rn}}{300~Bq.kg^{-1}} \end{aligned}$$

6 $$\begin{aligned}{} & {} H_{ex} = \frac{A_{Th}}{259} + \frac{A_{Ra}}{370} + \frac{A_{K}}{4810} = \frac{Ra_{eq}}{370~Bq.kg^{-1}}, \end{aligned}$$

where, $A_{Ra}$, $A_{Th}$ and $A_{K}$ are the specific activity concentrations of $^{226}Ra$, $^{232}Th$ and $^{40}K$ respectively.

#### Absorbed gamma dose rate (*D*)

The term “absorbed gamma dose rate” describes the radiation energy that a unit mass of material or a human body absorbs per unit of time as a result of exposure to gamma rays^53^. The unit of absorbed dose is the gray (Gy), defined as the absorption of one joule of radiation energy per kilogram of material. In addition, the absorbed gamma dose rate is the absorbed dose per unit of time, and its unit is the gray per hour (Gy h$^{-1}$). It measures the rate at which the material or the human body absorbs radiation energy^54,55^. The absorbed gamma dose rate depends on several factors, including the intensity of the gamma radiation, the distance from the radiation source, and the shielding materials that may be present. Therefore, the absorbed gamma dose rate is an essential parameter in radiation protection, as it assesses the potential health risks of exposure to gamma radiation. Exposure to high levels of gamma radiation can cause tissue damage and increase the risk of cancer and other radiation-related illnesses^56^. Hence, the absorbed dose rate can be assessed based on the following formula^57,58^:

7 $$\begin{aligned}{} & {} D_{out}(nGy.h^{-1}) = 0.604 \times A_{Th} + 0.462 \times A_{Ra} + 0.0417 \times A_{K} \end{aligned}$$

8 $$\begin{aligned}{} & {} D_{in}(nGy.h^{-1})= 1.1 \times A_{Th} + 0.92 \times A_{Ra} + 0.08 \times A_{K} \end{aligned}$$

where; $D_{out}$, is the absorbed gamma dose rate in the air at 1m overhead the ground level, and $D_{in}$ is the rate by which the dose will be received by a dweller or an occupant, assuming that he is living in a standard room made mostly from those investigated materials.

#### Outdoor and indoor annual effective dose ($E_{out}$/$E_{in}$)

The annual effective dose estimates the amount of radiation a person is exposed to during a year^59^. It is a beneficial concept to assess the potential risks associated with outdoor or indoor exposure to ionizing radiation. The annual effective dose depends on various factors, such as the type and amount of radiation, the duration and frequency of exposure, and the environment in which exposure occurs.

Outdoor annual effective dose ($E_{out}$) is the annual dose of ionizing radiation, especially $\gamma$-rays, that a person receives from natural sources such as cosmic radiation and naturally occurring radioactive materials in soil, water, and air. On the other hand, the indoor annual effective dose ($E_{in}$) is the annual dose of ionizing radiation that a person receives from natural or man-made sources, which are in the current study of the investigated minerals when staying indoors in a closed, improperly ventilated area, assuming that the dweller spends about 80$\%$ of its day in this closed area^60^. Artificial sources are building materials, consumer products such as smoke detectors, and medical procedures that use ionizing radiation. Radioactive radon gas, which results from uranium’s natural decay, is one of the natural sources of indoor radiation. The indoor annual effective dose varies depending on several factors, such as the type of building materials, ventilation quality, and the level of radon in the indoor environment. The following equations have been used to determine ($E_{out}$, and $E_{in}$) based on ($D_{out}$ and $D_{in}$) respectively^26,61^:

9 $$\begin{aligned}{} & {} E_{out}(mSv.y^{-1})=D_{out}(nGy.h^{-1})\times 8760(h.y^{-1}) \times 0.2(OF) \times 0.7(Sv.Gy^{-1})\times 10^{-6} \end{aligned}$$

10 $$\begin{aligned}{} & {} E_{in}(mSv.y^{-1})=D_{in}(nGy.h^{-1})\times 8760(h.y^{-1}) \times 0.8(OF)\times 0.7(Sv.Gy^{-1})\times 10^{-6} \end{aligned}$$

where OF is the occupancy factor that can be determined based on the time spent in the area, whether it is outdoor or indoor.

### Consent to participate

All authors agree to participate in the published version of the manuscript.

## Results and discussion

### Activity concentrations of the BSS samples

Table 2 presents the activity concentration values of $^{226}Ra$, $^{232}Th$ and $^{40}K$ in the collected BSS samples. The activity concentrations are given with uncertainties (standard deviations), reflecting the measurements’ precision.

**Table 2:** Activity concentration of $^{226}Ra$, $^{232}Th$ and $^{40}K$ for the BSS samples.

Sample code	Sample ID	Activity concentrations, (Bq Kg$^{-1}$)		
		$^{226}Ra$	$^{232}Th$	$^{40}K$
SS-1	Ore (1)	134.96±2.02	161.02±1.29	126.33±1.01
SS-2	Ore (2)	128.46±2.06	175.02±2.28	128.53±2.83
SS-3	Garnite	267.92±3.22	542.90±4.34	135.32±2.98
SS-4	Ilmenite	180.06±2.43	397.78±1.99	71.26±0.36
SS-5	Magnetite	80.57±1.05	98.93±0.40	65.01±0.65
SS-6	Sillica	350.16±4.20	771.78±2.32	70.49±0.14
SS-7	Rutile (1)	3305.95±82.65	3493.17±69.86	316.27±3.48
SS-8	Rutile (2)	4926.05±98.52	4124.80±16.50	310.27±1.55
SS-9	Zirrcon	4590.35±110.17	1683.10±3.30	181.05±0.54
SS-10	Monazite product (1)	2600.45±57.21	7062.27±176.56	602.75±7.23
SS-11	Monazite product (2)	2665.00±5.86	10351.07±217.37	501.58±6.17
SS-12	Monazite product (3)	3148.55±10.08	5803.67±203.13	378.29±4.92
WS-13	Wast lake	31.92±0.16	81.16±0.08	20.65±0.08
WS-14	Supply lake	21.92±0.22	20.49±0.12	22.65±0.11
WS-15	Shaking tables (1)	94.52±1.04	34.51±0.38	21.73±0.11
WS-16	Shaking tables (2)	111.92±0.25	40.49±0.40	80.65±0.40

The activity concentrations of three monazite samples SS(10-12) have been determined for $^{232}Th$, $^{226}Ra$, and $^{40}K$, yielding maximum values of 10351.10 ± 217.40, 3148.60 ± 10.10, and 602.80 ± 7.20 Bq Kg$^{-1}$ for $^{232}Th$, $^{226}Ra$ and $^{40}K$, respectively. While the activity concentrations for $^{232}Th$, $^{226}Ra$v and $^{40}K$ of the two rutile samples SS-7 and SS-8 show maximum values of 4124.80 ± 16.50 Bq Kg$^{-1}$ for $^{232}$Th, 4926.05 ± 98.52 Bq Kg$^{-1}$ for $^{226}$Ra, and 316.27 ± 3.48 Bq Kg$^{-1}$ for $^{40}K$. In addition, the activity concentrations of the zirrcon sample SS-9 represent significant high values for $^{232}Th$, $^{226}Ra$, and $^{40}K$ as presented in Table 2. The other activity concentrations of the other six samples (SS-1 to SS-6), which represent ore, granite, ilmenite, silica, and magnetite, show notably lower values considering the contents of the analyzed NORMs compared to the formerly discussed samples: monazite, rutile, and zircon. Additionally, the four WS, including shaking tables water, wastewater lake, and supply water lake samples WS (14–17), show the lowest activity concentration values, even below the global average values.

Overall, the results show that the activity concentrations of $^{232}Th$ and $^{226}Ra$ are generally higher in the mineral samples (monazite, rutile, and zircon) compared to the other investigated samples. The reasons for the former findings could be attributed to two main reasons: The first reason is the successive separation processes that minimize the amount of thorium, uranium, and potassium-bearing minerals in some separated products, such as ilmenite and magnetite; however, at the same time, these minerals are concentrated in the other separated products, especially monazite, rutile, and zircon. The other important reason is the geochemical nature of the separated products. For example, monazite is mainly composed of phosphate minerals; most of them are rare-earth elements’ phosphates beside thorium phosphate, usually (Ce, La, Nd, Th) PO$_{4}$, with other commonly attached minerals like potassium silicates. This can explain why monazite samples possess the highest activity concentrations for the three NORMs among the studied samples. The same can be observed with rutile, which comes in second place considering the total activity concentration measured and is considered one of the most important titanium-bearing heavy minerals. Other U-Th-bearing heavy minerals can be found attached to rutile, which usually leads to the observed high activity concentration, especially for $^{232}$Th and $^{226}$Ra and their associated decay products.

### Radiation hazard indices

**Figure 4:**
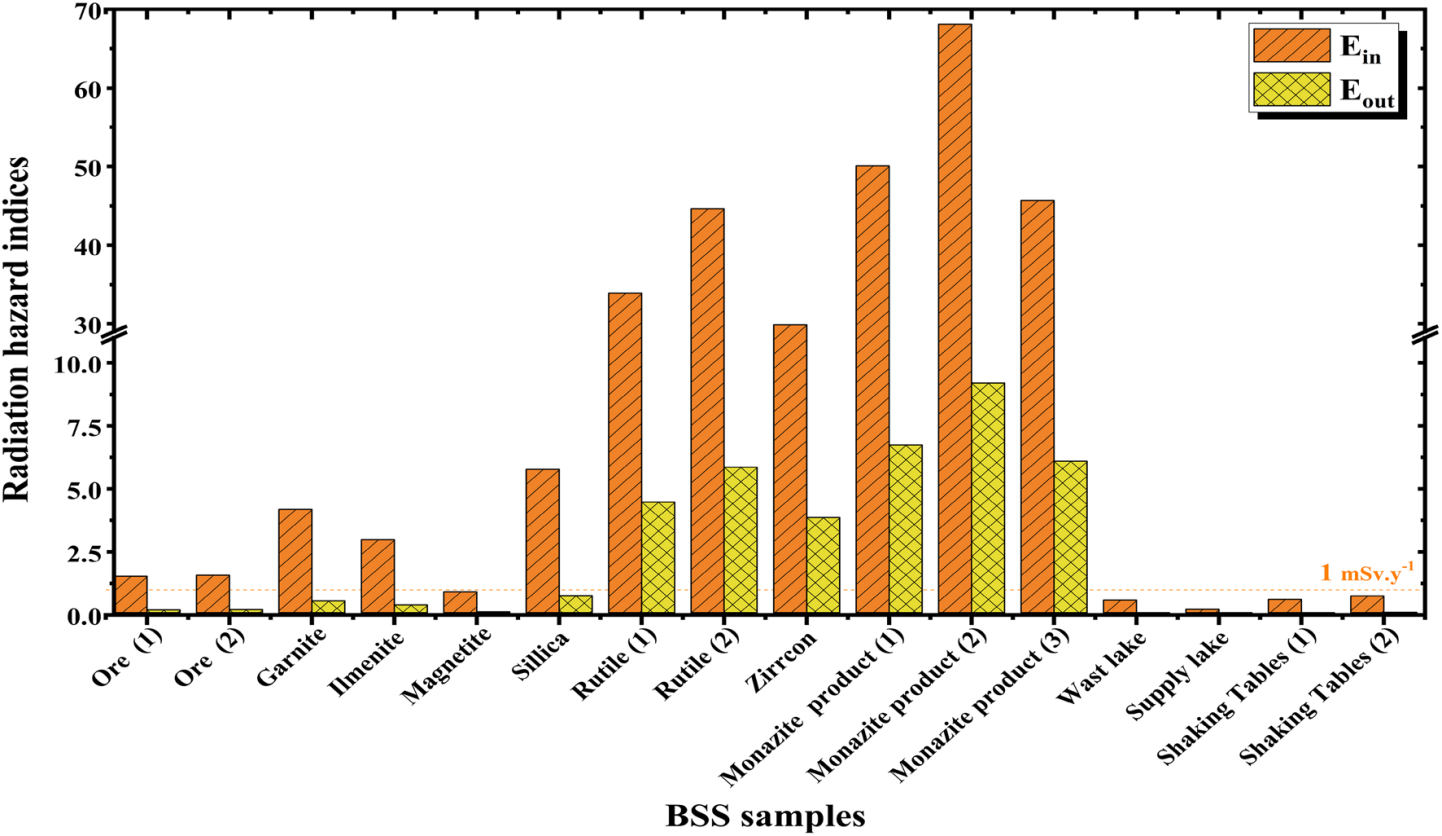
Indoor/Outdoor annual effective dose of the black sand separation samples.

**Figure 5:**
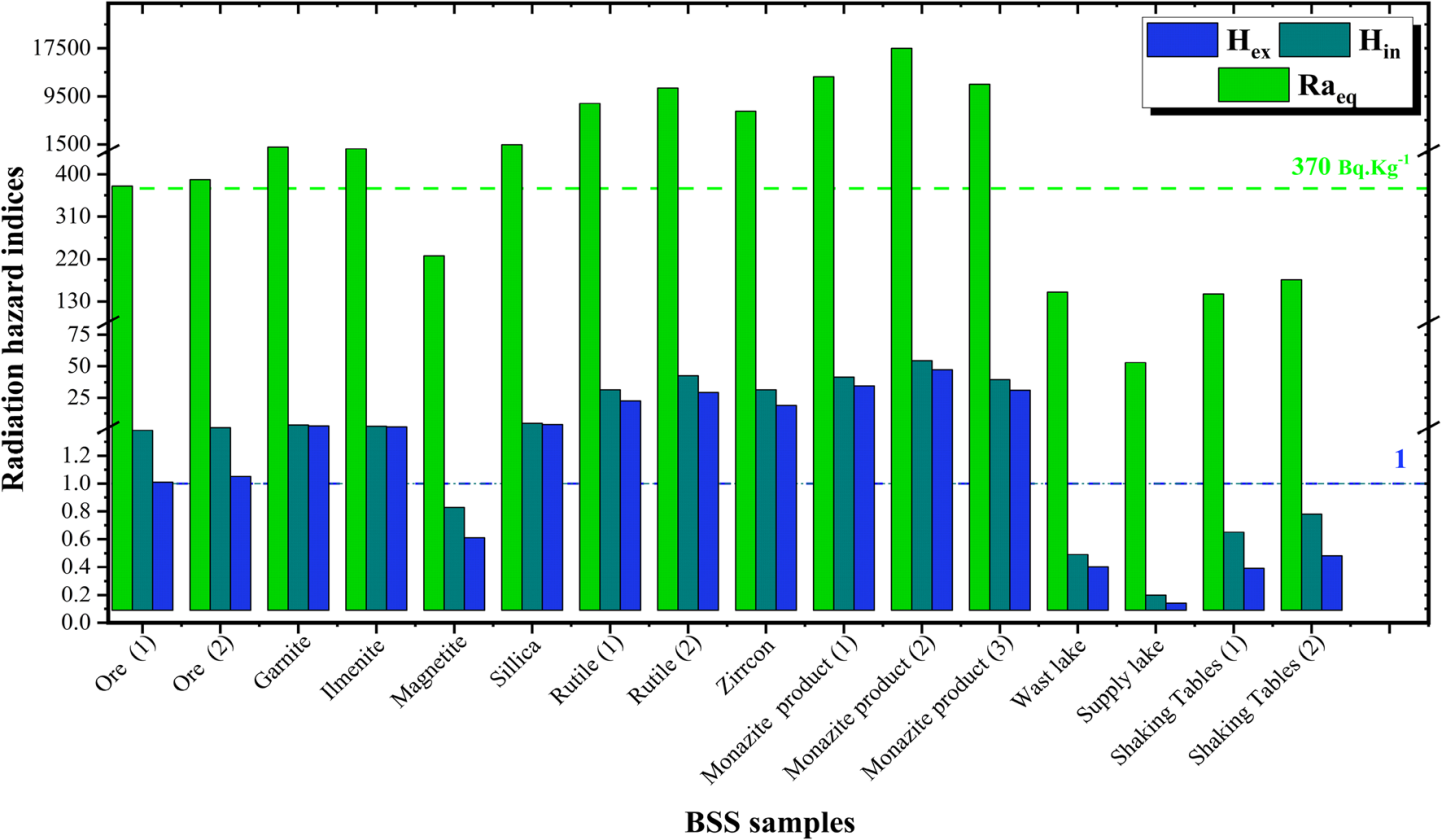
Radium equivalent and internal/external hazard index calculations of the investigated black sand separation samples.

Speaking of the external radiological hazard indices and the attributed dose first, all BSS samples except magnetite representing sample SS-5 show values greater than the recommended safe values, which are 370 Bq Kg$^{-1}$, 1, and 1 mSv y$^{-1}$ for Ra$_{eq}$, H$_{ex}$, and E$_{out}$, respectively^62^. Even the samples that represent the black sand ore before the separation process, SS-1, and SS-2, can show values slightly greater than those recommended safe values, as listed in Table 3 and illustrated in Figs. 4 and 5. Granite, ilmenite, and silica representing samples show values a few times higher than the safe values However, rutile, zircon, and monazite, representing samples SS-7 to SS-12, show significant risky values that are much higher than the recommended safe doses. For those three mineral products, Ra$_{eq}$, H$_{ex}$, and E$_{out}$ values range from 7011.12, 18.94, and 3.86, up to 17505.65, 47.27, and 9.20, respectively. The calculated external radiological hazard indices and the attributed dose show that there are external radiological risks that need to be protected for people who are in direct and long-term contact with the studied mineral black sand-derived products. In this study, this could include workers who mine, separate, and stack these products. These actions rely on the main three radiation protection principles^63^ as follows:

1. Reducing the exposure time, which can be achieved by increasing the on-site employment, the number of working shifts, and rotating the on-site workers among the different locations that possess different dose levels.
2. Increasing the separative distance between the person and the radiation source, which can be done by increasing the dependency on automated systems and equipment, especially in handling and piling up products like rutile, zircon, and monazite, which possess high levels of radioactivity due to their NORMs’ contents,
3. Proper radiation shielding, which can be applied by wearing suitable protective clothing that attenuates the emitted gamma rays from those materials, especially face masks, which are capable of greatly reducing the inhaled particulates from these minerals during the separation, handling, and piling up processes,.

On the other hand, considering the indoor radiological hazard indices and the attributed dose, the same trend and ranking observed while discussing the external radiological hazard indices can be observed here when analyzing the calculated indoor ones, except that the observed values are much higher.

**Table 3:** Results of radium equivalent, outdoor/indoor hazard index, and external/internal hazard index calculations of the investigated BSS samples.

Sample code	Sample ID	$D_{in}$ (nGy h$^{-1}$)	$E_{in}$ (mSv y$^{-1}$)	$D_{out}$ (nGy h$^{-1}$)	$E_{out}$ (mSv y$^{-1}$)	$Ra_{eq}$ (Bq Kg$^{-1}$)	$H_{in}$	$H_{ex}$
SS-1	Ore (1)	311.39	1.53	164.88	0.20	374.95	1.38	1.01
SS-2	Ore (2)	320.99	1.570	170.42	0.21	388.64	1.400	1.050
SS-3	Garnite	854.50	4.19	457.33	0.56	1054.69	3.57	2.85
SS-4	Ilmenite	608.91	2.99	326.42	0.40	754.37	2.52	2.04
SS-5	Magnetite	188.15	0.92	99.69	0.12	227.05	0.83	0.61
SS-6	Sillica	1176.74	5.77	630.87	0.77	1459.23	4.89	3.94
SS-7	Rutile (1)	6909.26	33.89	3650.41	4.48	8325.54	31.42	22.49
SS-8	Rutile (2)	9094.07	44.61	4780.15	5.86	10848.40	42.62	29.30
SS-9	Zirrcon	6089.02	29.87	3144.88	3.86	7011.12	31.35	18.94
SS-10	Monazite product (1)	10209.13	50.08	5492.15	6.74	12745.91	41.45	34.42
SS-11	Monazite product (2)	13878.10	68.08	7504.19	9.20	17505.65	54.48	47.27
SS-12	Monazite product (3)	9310.97	45.68	4975.82	6.10	11476.93	39.51	31.00
WS-13	Wast lake water	120.29	0.59	64.63	0.08	149.57	0.49	0.40
WS-14	Supply lake water	44.52	0.22	23.45	0.03	52.96	0.20	0.14
WS-15	Shaking tables water(1)	126.66	0.62	65.42	0.08	145.54	0.65	0.39
WS-16	Shaking tables water (2)	153.96	0.76	79.53	0.10	176.03	0.78	0.48
The recommended safe maximum limits^65^		–	1	–	1	370	1	1

For example, while the outdoor dose E$_{out}$ ranges from 0.2 (SS-1) to 9.2 (SS-11) mSv y$^{-1}$, the evaluated possible indoor dose E$_{in}$ has been found to range from 1.53 (SS-1) to 68.1 (SS-11) mSv y$^{-1}$. Before discussing the reason for that, and just for more clarification, the possible indoor dose/hazard indices are those experienced by dwellers or occupants who reside or spend much time indoors in closed, improperly ventilated buildings constructed mainly from materials containing bulk amounts of these studied materials. The doses, in that case, come from these materials due to their contents of NORMs directly through the walls without efficient shielding, except for some self-shielding as these materials are the main constituents of the rooms’ walls. That is why those studied BSS minerals should be avoided being used in construction applications, except for magnetite.

Returning to the indoor indices and their significantly higher values in comparison to the outdoor ones, the reason is mainly attributed to doubling the effects of the emitted radiation, especially from $^{226}$Ra and its associated decay products. Outdoors, the emitted gamma rays are the only radiation that is considered, while indoors, both gamma rays and alpha particles emitted from the accumulated radon, $^{222}$Rn, have to be considered while estimating the indoor hazard indices and the attributed indoor dose. Considering the collected water samples, which represent the waste lake, supply lake, and deposited water from shaking tables during the drying process, all four samples (WS-13 to WS-16) show safe outdoor and indoor values for hazard indices and the attributed doses, which ensure that the site surrounding water bodies hasn’t been significantly contaminated by the radionuclides existing in the studied black sand separated products during and after the separation process.

Finally, it is not a surprise that the estimated radiological hazard indices, whether outdoor or indoor, are all correlated to the measured activity concentrations, which magnify the consequences of the BSS separation processes and enlighten the importance of taking the necessary protective actions to protect either the on-site workers (20 mSv y$^{-1}$) or the public (1 mSv.y$^{-1}$) from the radiological risks attributed to prolonged exposure and dealing with these mineral products^64^.

## Conclusion

A radiological assessment of a black sand separation plant (in Delta) was carried out by investigating several samples collected from the plant’s production line and storage areas. Based on the activity levels measured from the NORMs naturally found in the materials being studied and the corresponding radiological hazard indices, the following conclusions can be drawn:

- The measured activity concentrations for all studied samples show that all the collected samples possess net specific radioactivity due to their contents of NORMs, higher than the maximum permissible safe limits except for magnetite representing the sample.
- The estimated outdoor radiological hazard indices calculated based on the measured activity concentrations were found to be higher than the recommended safe limits for all studied BSS samples except one sample, which indicates that all black sand-separated products and even the ore, except magnetite, can cause hazardous radiological risks for the on-site workers who are in prolonged exposure to these products.
- The indoor radiological hazard indices and the attributed doses for the studied samples reveal the significant radiological risks for the indoor occupants if these investigated materials are used in building residence places, again except magnetite, especially the three BSS products: zircon, rutile, and monazite. Thus, those materials cannot be used in manufacturing building materials or any construction applications except for magnetite.
- Based on the former, it becomes clear that the various black sand separation processes concentrate the NORMs in the separated products while decreasing their amounts in other separated products like magnetite.
- Last but not least, whether we consider the BSS workers on-site as workers in the radiation field or not, protective measures, as suggested in the study, must be taken to protect them from accumulated external and internal radiological doses, which may eventually lead to chronic effects like the risk of cancer, life-shortening, or mutations.

## Data Availability

All data generated or analyzed during this study is included in this published article.
